# *Pneumocystis jirovecii* Genotype Associated with Increased Death Rate of HIV-infected Patients with Pneumonia

**DOI:** 10.3201/eid1901.120140

**Published:** 2013-01

**Authors:** Meja Rabodonirina, Laetitia Vaillant, Patrick Taffé, Aimable Nahimana, René-Pierre Gillibert, Philippe Vanhems, Philippe M. Hauser

**Affiliations:** Author affiliations: Université Claude-Bernard Hôpital de la Croix-Rousse, Lyon, France (M. Rabodonirina);; Service d’Hygiène, Epidémiologie et Prévention, Hôpital Edouard Herriot, Lyon (L. Vaillant, R.-P. Gillibert, P. Vanhems);; Institut de Veille Sanitaire, Paris, France (L. Vaillant);; Centre Hospitalier Universitaire Vaudois and University of Lausanne, Lausanne, Switzerland (P. Taffé, A. Nahimana, P.M. Hauser);; Claude Bernard Lyon 1 University, Lyon (P. Vanhems)

**Keywords:** Pneumocystis jirovecii pneumonia, Pneumocystis jirovecii dihydropteroate synthase, DHPS, HIV, homosexuality, intravenous drug use, dihydropteroate synthase mutations, opportunistic infection, immunocompromised, virus, fungus, fungi, fungal, sulfa resistance, sulfamethoxazole/trimethoprim, SMX/TMP, dapsone, pentamidine, atovaquone, antimicrobial drugs, antibiotic, antifungal drugs

## Abstract

Comorbidities might predict presence of specific fungal genotypes.

## Medscape CME Activity

Medscape, LLC is pleased to provide online continuing medical education (CME) for this journal article, allowing clinicians the opportunity to earn CME credit. 

This activity has been planned and implemented in accordance with the Essential Areas and policies of the Accreditation Council for Continuing Medical Education through the joint sponsorship of Medscape, LLC and Emerging Infectious Diseases. Medscape, LLC is accredited by the ACCME to provide continuing medical education for physicians.

Medscape, LLC designates this Journal-based CME activity for a maximum of 1 *AMA PRA Category 1 Credit(s)*^TM^. Physicians should claim only the credit commensurate with the extent of their participation in the activity.

All other clinicians completing this activity will be issued a certificate of participation. To participate in this journal CME activity: (1) review the learning objectives and author disclosures; (2) study the education content; (3) take the post-test with a 70% minimum passing score and complete the evaluation at www.medscape.org/journal/eid; (4) view/print certificate.

**Release date: December 20, 2012; Expiration date: December 20, 2013**

## Learning Objectives

Upon completion of this activity, participants will be able to:

- Distinguish the rate of dihydropteroate synthase (DHPS) mutations among patients with *Pneumocystis jirovecii* pneumonia (PCP) in the current study
- Analyze patient characteristics associated with a higher rate of DHPS mutations
- Assess variables associated with sulfa resistance among cases of PCP in the current study
- Evaluate the effects of DHPS mutations on the risk of death among cases of PCP in the current study.

## CME Editor

**Jean Michaels Jones,** Technical Writer/Editor, Emerging Infectious Diseases. *Disclosure: Jean Michaels Jones has disclosed no relevant financial relationships.*

## CME Author

**Charles P. Vega, MD, **Health Sciences Clinical Professor; Residency Director, Department of Family Medicine, University of California, Irvine. *Disclosure: Charles P. Vega, MD, has disclosed no relevant financial relationships.*

## Authors

*Disclosures:* ***Meja Rabodonirina, MD, PhD*** *;* ***Laetitia Vaillant, MD*** *;* ***Patrick Taffé, PhD*** *;* ***Aimable Nahimana, PhD*** *;* ***René-Pierre Gillibert, PhD*** *; and* ***Philippe Hauser, PhD*** *, have disclosed no relevant financial relationships.* ***Philippe Vanhems, MD, PhD*** *, has disclosed the following relevant financial relationships: received grants for clinical research from Sanofi Pasteur.*
