# Supplementary material for: Pneumocystis jirovecii Genotype Associated with Increased Death Rate of HIV-infected Patients with Pneumonia
Source: Emerg Infect Dis. 2013 Jan;19(1):21–8. doi: 10.3201/eid1901.120140 (PMC3557975; doi:10.3201/eid1901.120140)
Supplement: Related texts

# Research article: *Pneumocystis jirovecii* Genotype Associated with Increased Death Rate of HIV-infected Patients with Pneumonia

**Authors:** Meja Rabodonirina, Laetitia Vaillant, Patrick Taffé, Aimable Nahimana, René-Pierre Gillibert, Philippe Vanhems, Philippe M. Hauser

**Affiliations:** Author affiliations: Université Claude-Bernard Hôpital de la Croix-Rousse, Lyon, France (M. Rabodonirina);; Service d’Hygiène, Epidémiologie et Prévention, Hôpital Edouard Herriot, Lyon (L. Vaillant, R.-P. Gillibert, P. Vanhems);; Institut de Veille Sanitaire, Paris, France (L. Vaillant);; Centre Hospitalier Universitaire Vaudois and University of Lausanne, Lausanne, Switzerland (P. Taffé, A. Nahimana, P.M. Hauser);; Claude Bernard Lyon 1 University, Lyon (P. Vanhems)

## Abstract

*Pneumocystis jirovecii* dihydropteroate synthase (DHPS) mutations have been associated with failure of sulfa prophylaxis; their effect on the outcome of patients with *P. jirovecii* pneumonia (PCP) remains controversial. *P. jirovecii* DHPS polymorphisms and genotypes were identified in 112 cases of PCP in 110 HIV-infected patients by using PCR single-strand conformation polymorphism. Of the 110 patients observed, 21 died; 18 of those deaths were attributed to PCP. Thirty-three percent of the PCP cases involved a *P. jirovecii* strain that had 1 or both DHPS mutations. The presence or absence of DHPS mutations had no effect on the PCP mortality rate within 1 month, whereas *P.jirovecii* type 7 and mechanical ventilation at PCP diagnosis were associated with an increased risk of death caused by PCP. Mechanical ventilation at PCP diagnosis was also associated with an increased risk of sulfa treatment failure at 5 days.

*Pneumocystis jirovecii* causes severe pneumonia in immunocompromised patients, including HIV-infected persons, transplant recipients, patients receiving high-grade chemotherapy for hemato-oncologic diseases, and persons with autoimmune diseases who are treated with immunosuppressive drugs. Cotrimoxazole, the combination of sulfamethoxazole and trimethoprim (SMX/TMP), is the drug of choice for prevention of and treatment for *Pneumocystis* pneumonia (PCP). SMX/TMP targets enzymes involved in the biosynthesis of folic acid, dihydropteroate synthase (DHPS), and dihydrofolate reductase.

Several investigators have reported an association between failure of prophylaxis when using sulfa drugs and substitutions of 2 aa within the putative sulfa binding site of DHPS at positions 55 (Thr to Ala, mutation M1) and 57 (Pro to Ser, M2) (*1**–**4*). These mutations were observed either as single (M1 or M2) or double (M3) mutation. This association strongly suggested that *P. jirovecii* DHPS mutations conferred a level of sulfa resistance sufficient to cause failure of anti-PCP prophylaxis. However, the mutations might have also conferred a clinically substantial resistance to sulfa treatment for overt PCP.

To investigate the issue, many studies have analyzed the effect of the mutations on the outcome of PCP. About half of those studies did not detect any association between the mutations and an increased risk of death caused by PCP (*5**–**8*) or a decreased response to sulfa drugs (*3**,**9**–**11*). Conversely, other studies detected an association with a poor outcome (*12**,**13*): sulfa treatment failure (*14**,**15*); more severe symptoms and need of assisted ventilation (*13*); or a trend for a worse prognosis (*16*). Thus, the effect of these mutations on PCP outcome is unclear and justifies investigation to improve PCP treatment and prognosis.

The possibility of other parameters influencing PCP outcome has also been explored. *P. jirovecii* genotype Ne of the internal transcribed spacers (ITSs) of the nuclear rRNA operon has been associated with milder disease (*17*), failure of PCP prophylaxis (*18*), and failure of PCP treatment (*9*). One ITS genotype observed in Australia was associated with reduced severity of PCP (*13*). Specific *P. jirovecii* genotypes defined by single-nucleotide polymorphisms in 3 loci were associated with low or high burden during the course of PCP (*19*). In comparison, some studies did not detect any associations between *P. jirovecii* genotypes, including Ne genotype, and several clinical parameters, such as severity and survival at 3 months (*15**,**20*). These observations suggested that some *P. jirovecii* genotypes might be more virulent or resistant to drugs, but further studies are needed to provide better understanding the issue.

We previously examined *P. jirovecii* DHPS polymorphisms in clinical specimens of 158 immunosuppressed patients from 5 hospitals in the city of Lyon in France (*7*). We detected an association between DHPS mutation M2 and failure of prophylaxis when pyrimethamine/sulfadoxine was used but not between DHPS mutations and death caused by PCP. In this study, we further observed the proportion of the organisms harboring DHPS mutations (36%) and of death attributed to PCP (20%) among these 158 patients. We investigated in more detail the effect of DHPS mutations on PCP prognoses, taking into account more clinical parameters. Moreover, to test the hypothesis of variable virulence of some *P. jirovecii* genotypes, we identified those present in the specimens. Because the disease signs and symptoms vary considerably between HIV-infected and HIV-uninfected patients, we limited our analyses to the HIV-infected patients.

## Patients, Materials, and Methods

### Patients and Specimens

The specimens consisted of 112 bronchoalveolar lavage (BAL) samples obtained from 110 HIV-infected patients with confirmed PCP who were hospitalized in 5 university hospitals in Lyon, France. These 110 patients were a subset of the 158 patients analyzed (*7*) who were HIV-infected and who had a medical chart complete enough to support the analyses performed in the present study (Tables 1, 2). Two of the patients had second cases of PCP separated from their initial infections by 5 and 12 months, respectively; each was treated as an independent observation. BAL specimens were collected during April 1993 and December 1996 and were stored at –20°C before analysis. The 112 cases represented 47% of the PCP cases that occurred during this period in the 5 hospitals.

**Table 1:** *Pneumocystis jiroveci* dihydropteroate synthase genotype distribution according to clinical parameters of 112 cases of pneumonia in 110 HIV-positive patients from 5 university hospitals in Lyon, France*

Characteristic	DHPS genotype	p value

Wild type, n = 75	M2, n = 17	M3, n = 20
Age at PCP diagnosis, y				
1–40	41	10	14	0.62
41–60	31	7	5	
61–80	3	0	1	
Sex				
M	62	14	19	0.38
F	13	3	1	
Diagnosis year				
1993	6	2	0	0.58
1994	24	3	4	
1995	20	5	6	
1996	25	7	10	
CD4 cell count, median cells/μL†				
0–50	42	15	11	0.05
51–100	17	0	4	
>100	10	0	2	
First-line treatment				
SMX/TMP	59	14	15	0.90
Pentamidine or atovaquone	14	3	5	
Others	2	0	0	
SMX/TMP allergy				
Yes	4	0	3	0.27
No	71	17	17	
Corticotherapy at PCP diagnosis				
Yes	18	1	3	0.25
No	57	16	17	
Mechanical ventilation at PCP diagnosis				
Yes	11	0	3	0.26
No	64	17	17	
*P. jirovecii* SSCP type 1	3	6	1	<0.001
7	3	0	6	
10	2	1	1	
Others	30	3	3	
Co-infected	37	7	9	
Outcome within 1 month				
Favorable	61	16	14	0.39
Death attributed to PCP	12	1	5	
Death not attributed to PCP	2	0	1	

*Data derived from Fisher’s exact test. DHPS, dihydropteroate synthase; M, mutation; SSCP, single-strand conformation polymorphism; PCP, *P. jirovecii* pneumonia; SMX/TMP, sulfamethoxazole and trimethoprim.  †Data missing for 11 patients.

**Table 2:** *Pneumocystis jiroveci* SSCP genotype distribution according to clinical parameters of 112 cases of pneumonia in 110 HIV-positive patients from 5 university hospitals in Lyon, France*

Characteristic	*P. jirovecii* SSCP type	p value

1, n = 10	7, n = 9	10, n = 4	Others, n = 36	Co-infected, n = 53
Age at PCP diagnosis, y						
1–40	8	5	2	22	28	0.89
41–60	2	4	2	13	22	
61–80	0	0	0	1	3	
Sex						
M	3	1	1	6	6	0.44
F	7	8	3	30	47	
Diagnosis year						
1993	1	0	0	2	5	0.90
1994	3	2	2	8	16	
1995	2	2	1	9	17	
1996	4	5	1	17	15	
CD4 cell count, median cells/μL†						
0–50	7	5	3	23	30	0.87
51–100	0	2	1	6	12	
>100	1	1	0	5	5	
First-line treatment						
SMX/TMP	7	3	3	26	49	0.001
Pentamidine or atovaquone	2	6	1	9	4	
Others	1	0	0	1	0	
SMX/TMP allergy						
Yes	0	2	0	3	2	0.27
No	10	7	4	33	51	
Corticotherapy at PCP diagnosis						
Yes	0	4	0	7	11	0.17
No	10	5	4	29	42	
Mechanical ventilation at PCP diagnosis						
Yes	0	2	0	2	10	0.19
No	10	7	4	34	43	
Outcome within 1 month						
Favorable	9	5	4	28	45	0.44
Death attributed to PCP	1	4	0	6	7	
Death not attributed to PCP	0	0	0	2	1	

*Data derived from Fisher’s exact test. SSCP, single-strand conformation polymorphism; PCP, *P. jirovecii* pneumonia; SMX/TMP, sulfamethoxazole and trimethoprim.  †Data missing for 11 patients.

### Characteristics of Patients

Specific information on demographic and clinical characteristics, treatment regimens, and PCP outcome were obtained from patients’ medical charts. Death within 1 month after the date of PCP diagnosis was attributed to PCP when the physician recorded it as the primary cause of death in the medical chart and on the death certificate. Failure of sulfa treatment (SMX/TMP or dapsone) was defined by occurrence of >1 of the following events within 5 days after PCP diagnosis: a change of drug treatment because of lack of clinical improvement, worsening of clinical features or gas exchange parameters, addition of corticosteroids, new need of mechanical ventilation, or death attributed to PCP. Start-of-therapy dates were available for 81 patients. Therapy was started during 1 of 3 periods: before the day on which PCP was diagnosed (n = 34, 42%); the day on which PCP was diagnosed (n = 37, 46%); or after that day (n = 10, 12%). Therapy was started most frequently 1 or 2 days before the day on which BAL was obtained (n = 26, 77% of the 34 patients whose therapy was started before PCP was diagnosed). All chart abstractions were performed without knowledge of *P. jirovecii* and DHPS genotyping results. Informed consent was obtained from all patients. Study protocols and patient consent forms were approved by the institutional review board.

### DHPS and Genotyping

DNA extraction from BAL specimens and DHPS binding site genotyping by using the PCR–single-strand conformation polymorphism (SSCP) technique were done as described (*7*). Four DHPS SSCP patterns were observed, each corresponding to 1 of the 4 known DHPS alleles (M1, M2, M3, and wild type). *P. jirovecii* present in BAL specimens were typed as described by using PCR-SSCP of 4 variable genomic regions (*21*). The variable regions analyzed were ITS1 of the nuclear rDNA operon, the intron of the nuclear 26S rRNA gene, the variable region of the mitochondrial 26S rRNA gene, and the region surrounding the intron no. 6 of the β-tubulin gene. In the PCR-SSCP technique, each allele is identified by a specific SSCP pattern made of 2 DNA bands, each corresponding to 1 of the 2 single strands of the allele.

### Statistical Analysis

Because of small sample sizes, we used Fisher exact tests for general association to compare proportions relative to the numbers presented in Tables 1 and 2. We estimated the 2-month survival curves after PCP by using the Kaplan-Meier method and compared those curves by using log-rank tests. Cox proportional hazards method was used to identify independent prognostic factors associated with survival. The risk factors were selected a priori based on theoretical considerations; because no deaths occurred among women, we did not include gender in the variables. Also, the variable CD4 cell count was not included because it had 1) numerous missing values (n = 11, including 3 deaths attributed to PCP), and 2) no significant association with death attributed to PCP in bivariate analysis (p = 0.09). We used logistic regression analysis to identify factors associated with sulfa treatment failure; odds ratios with 95% CIs are reported. We tested the proportionality assumption using the nonzero slope test based on the scaled Schoenfeld residuals, and checked the colinearity (mean variance inflation factor = 1.16). All statistical analyses were conducted by using STATA version 11.1 (StataCorp LP, College Station, TX, USA).

## Results

### Specimens and Patients

One hundred twelve BAL specimens collected from 110 HIV-infected patients in whom PCP was diagnosed were included in the study. Two of the patients had second cases of PCP. Two patients also had tuberculosis, and 1 had histoplasmosis; each of the 3 recovered from PCP. The cohort ranged in age from 4–69 years (median 37 years); 94 (85%) were men. The most common risk factor for HIV was homosexuality (41%); the next most common risk factor was intravenous drug use (8%). CD4 counts at the diagnosis of PCP were documented in the medical charts of 101 patients and ranged from 0 to 390/μL (median 24). In 2 cases, patients had CD4 cell counts >200 cells/µL (242 and 390).

### Detection of DHPS Alleles by using PCR-SSCP

We previously genotyped the *P. jirovecii* DHPS binding site from the 112 BAL specimens by PCR amplification of a region of 318 bp, then SSCP (*7*). To simplify the analyses, specimens that contained a mixture of wild type and mutant DHPS genotypes (n = 11) were classified in the corresponding mutant category. For most of the cases, the patients harbored *P. jirovecii* with wild type DHPS (n = 75, 67.0%). Seventeen (15.2%) episodes involved a M2 mutant DHPS genotype, and 20 (17.8%) involved a M3 allele. The overall proportion of cases in which the patient had mutant DHPS was 33.0% (n = 37). There was a variation of this proportion from 25% to 57% among the 5 hospitals, but the difference did not reach statistical significance (*7*). No significant differences (p = 0.05) were found between patients who harbored *P. jirovecii* with DHPS mutations and those who did not for all the demographic or clinical characteristics analyzed.

### Genotyping by Using PCR-SSCP

Twenty-seven *P. jirovecii* genotypes were identified among the 112 BAL specimens by using the multitarget PCR-SSCP typing method (*21*). Fifty-nine (52.7%) specimens contained a single *P. jirovecii* type, 47 (42.0%) specimens contained 2 types, and 6 (5.3%) specimens contained more than 2 types that could not be identified. The 5 most prevalent genotypes were type 1 (n = 29 occurrences), type 7 (n = 15), type 10 (n = 12), type 2 (n = 10), and type 6 (n = 10). The 2 patients who had 2 cases each of PCP were infected with different types for each case, suggesting possible de novo infection for each case, rather than reactivation of the type that caused the first case.

### Associations between DHPS and *P. jirovecii* SSCP Genotypes and between Each Genotype and Clinical Factors 

Possible associations between DHPS and *P. jirovecii* SSCP genotypes and between each DHPS and *P. jirovecii* SSCP genotype and the clinical factors were investigated for the 112 PCP cases (Tables 1, 2). Specimens co-infected with >2 *P. jirovecii* SSCP types were gathered into a specific category, and the 3 most prevalent types among specimens containing a single genotype were considered separately. The sample was stratified into 5 groups: type 1, type 7, type 10, other types, and co-infected specimens. The DHPS mutations were not distributed evenly among the *P. jirovecii* SSCP type (p<0.001, by Fisher exact test) (Table 1), type 7 being associated with genotype M3 (66.7% of the types 7). The DHPS mutations were also unevenly distributed among the CD4 cell count groups (p = 0.05); mutation M2 was associated with <50 cells/μL (100%). Finally, the first-line treatment varied significantly according to the *P. jirovecii* SSCP type (p = 0.001) (Table 2), type 7 being most often treated with pentamidine or atovaquone rather than SMX/TMP (66.7%).

### Predictors of Death Attributed to PCP 

Of the 112 observed cases in this study, 21 (18.8%) patients died within 1 month. PCP was identified as the cause of death for 18 patients. Because of the small number of observations, the categories *P. jirovecii* type 10 and others types were further grouped with co-infected specimens, forming 3 groups: type 1, type 7, and other types. Bivariate analyses revealed that *P. jirovecii* SSCP type 7 and the need for mechanical ventilation at PCP diagnosis were possible predictors of death attributed to PCP (log-rank test, p = 0.08 and p<0.0001, respectively) (Figure). No effect of the DHPS mutated alleles on the PCP mortality rate was observed within 1 month (p = 0.35). Because type 7 was associated with DHPS mutation M3 and with first-line treatment with pentamidine or atovaquone, we included these variables in a multivariable analysis (Table 3). This analysis showed that type 7 and the need for mechanical ventilation at PCP diagnosis were significantly associated with an increased risk of death caused by PCP (relative hazard = 4.2, 95% CI 1.0–17.9, p = 0.05, and relative hazard = 5.0, 95% CI 1.8–13.5, p = 0.002, respectively) (Table 3). Similar results were obtained when the DHPS genotype variable was removed from the model.

**Figure:** Kaplan-Meier survival plots for 4 variables of 112 *Pneumocystis* pneumonia cases. A) p = 0.08; B) p = 0.35; C) p = 0.60; D) p = 0.0001. DHPS, dihydropteroate synthase.

**Table 3:** Multivariate analysis of risk factors for death attributed to PCP among 112 cases in 110 patients from 5 university hospitals, Lyon, France

Risk factor	Death attributed to PCP		Adjusted RH (95% CI)†	p value
	Yes	No		
*P. jirovecii* PCR-SSCP type				
7	4	5	4.2 (1.0–17.9)	0.05
Others	14	89	1.0 (Reference)	
Mechanical ventilation at PCP diagnosis				
Yes	8	6	5.0 (1.8–13.5)	0.002
No	10	88	1.0 (Reference)	
DHPS genotype				
M3	5	15	1.1 (0.4–3.4)	0.82
M2	1	16	0.6 (0.08–5.1)	0.66
Wild type	12	63	1.0 (Reference)	
Mean age at PCP diagnosis, y	38.5	40.6	1.0	0.95
First-line treatment				
Others	3	19	0.4 (0.1–2.0)	0.29
Sulfonamides	15	75	1.0 (Reference)	

*PCP, *Pneumocystis jirovecii* pneumonia; RH, relative hazard; SSCP, single-strand conformation polymorphism; DHPS, dihydropteroate synthase; M, mutation. †RH for death attributed to PCP after adjustment for the 5 variables presented in the table. The global p value of the test for proportionality was 0.55, and no individual test was statistically significant at the 0.05 level.

### Predictors of Sulfa Drug Treatment Failure 

To treat most cases, the patients received a sulfa drug as first-line treatment: for 88 (78.6%) cases, patients were treated with SMX/TMP, and for 2 (1.8%) cases, patients were treated with dapsone. Adverse effects of SMX/TMP in 5 patients led to a therapy change that excluded them from the analysis. Among the remaining 85 cases, 30 patients (35.3%) did not respond to the sulfa treatment at 5 days. Multivariate analysis of the same variables as for the analysis of predictors of death caused by PCP, except the first-line treatment, showed that an increased risk of failure of sulfa treatment was associated with the need for mechanical ventilation at PCP diagnosis (odds ratio 34.2, 95% CI 3.6–321.3, p = 0.002).

## Discussion

Analysis of the parameters of 112 PCP cases in HIV-infected patients involving a high proportion of *P. jirovecii* DHPS mutations and deaths attributed to PCP did not show an association between these mutations and a worse PCP outcome. This observation contrasts with studies that reported some negative influence of these mutations on PCP prognosis (*12**–**15*) but converges with results of other studies reporting no effect (*3**,**5**–**11*). The lack of standardization of the outcome parameters analyzed in the different studies, the confounders used for adjustment, and the small number of cases in some investigations might explain these conflicting conclusions. Nevertheless, a strong effect would have been identified even in studies with restricted sample sizes, suggesting that if these mutations have any effect on PCP outcome, it is small. Studies in the model organisms *Saccharomyces cerevisiae* (*22*) and *Escherichia coli* (*23*) strongly suggested that DHPS mutations confer some level of sulfa resistance to *P. jirovecii*. However, these reports did not speculate whether this level is sufficient to provoke clinical failure of sulfa treatment.

*P. jirovecii* type 7 was independently associated (i.e., nonnull partial correlation) with an increased risk for death caused by PCP. However, this type was also associated with DHPS mutation M3 (66.7% of the single infections), suggesting that the combination of the 2 parameters might have been necessary for its pathogenicity, even if mutation M3 alone was not a predictor of death caused by PCP. Consequently, we cannot exclude the role of mutation M3 for the increased virulence of *P. jirovecii* PCR-SSCP type 7. Type 7 was also associated with first-line treatment with pentamidine or atovaquone rather than SMX/TMP, suggesting that use of these less effective drugs might have contributed to an increased number of deaths of patients infected with type 7. However, the proportion of deaths attributed to PCP among these patients was higher with SMX/TMP than with pentamidine or atovaquone (2 of 3 vs. 2 of 6 patients, respectively). *P. jirovecii* PCR-SSCP type 7 has not been reported to have a higher virulence than the other types. *P. jirovecii* ITSs type B2a1, as well as other genotypes, were suggested to have higher and modified virulence (*9**,**13**,**17**–**19*). 

We observed that the need for mechanical ventilation at PCP diagnosis was associated with an increased risk for death caused by PCP, as was failure of sulfa treatment. An association of this comorbidity factor with an increased risk for death has already been reported (*24**,**25*). Several other clinical parameters were reported to be predictors for death caused by PCP in HIV-infected patients (*26*), and a scoring tool was recently proposed (*27*). Host polymorphisms within receptors involved in the immune response have also been reported to be related to *P. jirovecii* infection (*28**,**29**).* This study confirms that certain *P. jirovecii* genotypes might have different pathogenic traits. Further studies of PCR-SSCP type 7 could help understanding of *P. jirovecii* virulence and drug resistance by clinicians and public health professionals.

## References

[1] Mei Q, Gurunathan S, Masur H, Kovacs JA. Failure of co-trimoxazole in *Pneumocystis carinii* infection and mutations in dihydroperoate synthase gene. Lancet. 1998;351:1631–2 and. 10.1016/S0140-6736(05)77687-X9620722

[2] Kazanjian P, Locke AB, Hossler PA, Lane BR, Bartlett MS, Smith JW, *Pneumocystis carinii* mutations associated with sulfa and sulfone prophylaxis failures in AIDS patients. AIDS. 1998;12:873–8 and. 10.1097/00002030-199808000-000099631140

[3] Kazanjian P, Armstrong W, Hossler PA, Burman W, Richardson J, Lee CH, *Pneumocystis carinii* mutations are associated with duration of sulfa or sulfone prophylaxis exposure in AIDS patients. J Infect Dis. 2000;182:551–7 and. 10.1086/31571910915088

[4] Huang L, Crothers K, Atzori C, Benfield T, Miller R, Rabodonirina M, Dihydropteroate synthase gene mutations in *Pneumocystis* and sulfa resistance. Emerg Infect Dis. 2004;10:1721–8 and. 10.3201/eid1010.03099415504256PMC3323267

[5] Navin TR, Beard CB, Huang L, del Rio C, Lee S, Pieniazek NJ, Effect of mutations in *Pneumocystis carinii* dihydropteroate synthase gene on outcome of *P. carinii* pneumonia in patients with HIV-1: a prospective study. Lancet. 2001;358:545–9 and. 10.1016/S0140-6736(01)05705-111520525

[6] Ma L, Kovacs JA, Cargnel A, Valerio A, Fantoni G, Atzori C. Mutations in the dihydropteroate synthase gene of human-derived *Pneumocystis carinii* isolates from Italy are infrequent but correlate with prior sulfa prophylaxis. J Infect Dis. 2002;185:1530–2 and. 10.1086/34022011992293

[7] Nahimana A, Rabodonirina M, Zanetti G, Meneau I, Francioli P, Bille J, Association between a specific *Pneumocystis jiroveci* dihydropteroate synthase mutation and failure of pyrimethamine/sulfadoxine prophylaxis in human immunodeficiency virus-positive and -negative patients. J Infect Dis. 2003;188:1017–23 and. 10.1086/37823914513422

[8] Alvarez-Martínez MJ, Moreno A, Miró JM, Valls ME, Rivas PV, de Lazzari E, *Pneumocystis jirovecii* pneumonia in Spanish HIV-infected patients in the combined antiretroviral therapy era: prevalence of dihydropteroate synthase mutations and prognostic factors of mortality. Diagn Microbiol Infect Dis. 2008;62:34–43 and. 10.1016/j.diagmicrobio.2008.04.01618554841

[9] Matos O, Lee CH, Jin S, Li B, Costa MC, Gonçalves L, *Pneumocystis jiroveci* in Portuguese immunocompromised patients: association of specific ITS genotypes with treatment failure, bad clinical outcome and childhood. Infect Genet Evol. 2003;3:281–5 and. 10.1016/S1567-1348(03)00092-314636689

[10] Alvarez-Martínez MJ, Miró JM, Valls ME, Mas J, de la Bellacasa JP, Sued O, Prevalence of dihydropteroate synthase genotypes before and after the introduction of combined antiretroviral therapy and their influence on the outcome of *Pneumocystis* pneumonia in HIV-1-infected patients. Diagn Microbiol Infect Dis. 2010;68:60–5 and. 10.1016/j.diagmicrobio.2010.04.00720727472

[11] Visconti E, Ortona E, Mencarini P, Margutti P, Marinaci S, Zolfo M, Mutations in dihydropteroate synthase gene of *Pneumocystis carinii* in HIV patients with *Pneumocystis carinii* pneumonia. Int J Antimicrob Agents. 2001;18:547–51 and. 10.1016/S0924-8579(01)00460-511738342

[12] Helweg-Larsen J, Benfield TL, Eugen-Olsen J, Lundgren JD, Lundgren B. Effect of mutations in *Pneumocystis carinii* dihydropteroate synthase gene on outcome of AIDS-associated *P. carinii* pneumonia. Lancet. 1999;354:1347–51 and. 10.1016/S0140-6736(99)03320-610533864

[13] van Hal SJ, Gilgado F, Doyle T, Barratt J, Stark D, Meyer W, Clinical significance and phylogenetic relationship of novel Australian *Pneumocystis jirovecii* genotypes. J Clin Microbiol. 2009;47:1818–23 and. 10.1128/JCM.02102-0819369437PMC2691111

[14] Takahashi T, Hosoya N, Endo T, Nakamura T, Sakashita H, Kimura K, Relationship between mutations in dihydropteroate synthase of *Pneumocystis carinii* f. sp. *hominis* isolates in Japan and resistance to sulfonamide therapy. J Clin Microbiol. 2000;38:3161–4 .1097035010.1128/jcm.38.9.3161-3164.2000PMC87344

[15] Valerio A, Tronconi E, Mazza F, Fantoni G, Atzori C, Tartarone F, Genotyping of *Pneumocystis jiroveci* pneumonia in Italian AIDS patients. Clinical outcome is influenced by dihydropteroate synthase and not by internal transcribed spacer genotype. J Acquir Immune Defic Syndr. 2007;45:521–8 and. 10.1097/QAI.0b013e3180decbe217558331

[16] Crothers K, Beard CB, Turner J, Groner G, Fox M, Morris A, Severity and outcome of HIV-associated *Pneumocystis* pneumonia containing *Pneumocystis jirovecii* dihydropteroate synthase gene mutations. AIDS. 2005;19:801–5 and. 10.1097/01.aids.0000168974.67090.7015867494

[17] Miller RF, Wakefield AE. *Pneumocystis carinii* genotypes and severity of pneumonia. Lancet. 1999;353:2039–40 and. 10.1016/S0140-6736(99)01690-610376625

[18] Hauser PM, Sudre P, Nahimana A, Francioli P; Study Group. Prophylaxis failure is associated with a specific *Pneumocystis carinii* genotype. Clin Infect Dis. 2001;33:1080–2 and. 10.1086/32265911528584

[19] Esteves F, Gaspar J, De Sousa B, Antunes F, Mansinho K, Matos O. Clinical relevance of multiple single-nucleotide polymorphisms in *Pneumocystis jirovecii* pneumonia: development of a multiplex PCR-single-base-extension methodology. J Clin Microbiol. 2011;49:1810–5 and. 10.1128/JCM.02303-1021389160PMC3122690

[20] Helweg-Larsen J, Lee CH, Jin S, Hsueh JY, Benfield TL, Hansen J, Clinical correlation of variations in the internal transcribed spacer regions of rRNA genes in *Pneumocystis carinii* f.sp. *hominis.* AIDS. 2001;15:451–9 and. 10.1097/00002030-200103090-0000311242141

[21] Nahimana A, Blanc DS, Francioli P, Bille J, Hauser PM. Typing of *Pneumocystis carinii* f. sp. *hominis* by PCR-SSCP to indicate a high frequency of co-infections. J Med Microbiol. 2000;49:753–8 .1093326210.1099/0022-1317-49-8-753

[22] Meneau I, Sanglard D, Bille J, Hauser PM. *Pneumocystis jiroveci* dihydropteroate synthase polymorphisms confer resistance to sulfadoxine and sulfanilamide in *Saccharomyces cerevisiae.* Antimicrob Agents Chemother. 2004;48:2610–6 and. 10.1128/AAC.48.7.2610-2616.200415215117PMC434158

[23] Iliades P, Meshnick SR, Macreadie IG. Mutations in the *Pneumocystis jirovecii* DHPS gene confer cross-resistance to sulfa drugs. Antimicrob Agents Chemother. 2005;49:741–8 and. 10.1128/AAC.49.2.741-748.200515673759PMC547354

[24] Miller RF, Allen E, Copas A, Singer M, Edwards SG. Improved survival for HIV infected patients with severe *Pneumocystis jirovecii* pneumonia is independent of highly active antiretroviral therapy. Thorax. 2006;61:716–21 and. 10.1136/thx.2005.05590516601092PMC2104703

[25] Radhi S, Alexander T, Ukwu M, Saleh S, Morris A. Outcome of HIV-associated *Pneumocystis* pneumonia in hospitalized patients from 2000 through 2003. BMC Infect Dis. 2008;8:118 and. 10.1186/1471-2334-8-11818796158PMC2551597

[26] Wang HW, Lin CC, Kuo CF, Liu CP, Lee CM. Mortality predictors of *Pneumocystis jirovecii* pneumonia in human immunodeficiency virus-infected patients at presentation: experience in a tertiary care hospital of northern Taiwan. J Microbiol Immunol Infect. 2011;44:274–81 and. 10.1016/j.jmii.2010.08.00621524964

[27] Armstrong-James D, Copas AJ, Walzer PD, Edwards SG, Miller RF. A prognostic scoring tool for identification of patients at high and low risk of death from HIV-associated *Pneumocystis jirovecii* pneumonia. Int J STD AIDS. 2011;22:628–34 and. 10.1258/ijsa.2011.01104022096046

[28] Forthal DN, Landucci G, Bream J, Jacobson LP, Phan TB, Montoya B. FcgammaRIIa genotype predicts progression of HIV infection. J Immunol. 2007;179:7916–23 .1802523910.4049/jimmunol.179.11.7916

[29] Duggal P, An P, Beaty TH, Strathdee SA, Farzadegan H, Markham RB, Genetic influence of CXCR6 chemokine receptor alleles on PCP-mediated AIDS progression among African Americans. Genes Immun. 2003;4:245–50 and. 10.1038/sj.gene.636395012761559

# Research article: *Pneumocystis jirovecii* Genotype Associated with Increased Death Rate of HIV-infected Patients with Pneumonia

**Authors:** Meja Rabodonirina, Laetitia Vaillant, Patrick Taffé, Aimable Nahimana, René-Pierre Gillibert, Philippe Vanhems, Philippe M. Hauser

**Affiliations:** Author affiliations: Université Claude-Bernard Hôpital de la Croix-Rousse, Lyon, France (M. Rabodonirina);; Service d’Hygiène, Epidémiologie et Prévention, Hôpital Edouard Herriot, Lyon (L. Vaillant, R.-P. Gillibert, P. Vanhems);; Institut de Veille Sanitaire, Paris, France (L. Vaillant);; Centre Hospitalier Universitaire Vaudois and University of Lausanne, Lausanne, Switzerland (P. Taffé, A. Nahimana, P.M. Hauser);; Claude Bernard Lyon 1 University, Lyon (P. Vanhems)

## Medscape CME Quiz

## Earning CME Credit

To obtain credit, you should first read the journal article. After reading the article, you should be able to answer the following, related, multiple-choice questions. To complete the questions (with a minimum 70% passing score) and earn continuing medical education (CME) credit, please go to www.medscape.org/journal/eid. Credit cannot be obtained for tests completed on paper, although you may use the worksheet below to keep a record of your answers. You must be a registered user on Medscape.org. If you are not registered on Medscape.org, please click on the New Users: Free Registration link on the left hand side of the website to register. Only one answer is correct for each question. Once you successfully answer all post-test questions you will be able to view and/or print your certificate. For questions regarding the content of this activity, contact the accredited provider, CME@medscape.net. For technical assistance, contact CME@webmd.net. American Medical Association’s Physician’s Recognition Award (AMA PRA) credits are accepted in the US as evidence of participation in CME activities. For further information on this award, please refer to http://www.ama-assn.org/ama/pub/category/2922.html. The AMA has determined that physicians not licensed in the US who participate in this CME activity are eligible for *AMA PRA Category 1 Credits*™. Through agreements that the AMA has made with agencies in some countries, AMA PRA credit may be acceptable as evidence of participation in CME activities. If you are not licensed in the US, please complete the questions online, print the certificate and present it to your national medical association for review.

## Article Title:  *Pneumocystis jirovecii* Genotype Associated with Increased Death Rate of HIV-infected Patients with Pneumonia

## CME Questions

**1. You are seeing a 30-year-old man with HIV infection and a new diagnosis of *Pneumocystis jirovecii* pneumonia (PCP). You are concerned regarding the possibility of genetic mutations in this organism. What was the overall rate of dihydropteroate synthase (DHPS) mutations among cases of PCP in the current study?**

A. 4%

B. 33%

C. 74%

D. 83%

**2. DHPS mutations were most associated with which of the following patient characteristics in the current study?**

A. CD4 count less than 50 cells/μL

B. Age over 40 years

C. Male sex

D. Diagnosis of PCP after 1995

**3. You initiate treatment with trimethoprim-sulfamethoxazole. Which of the following variables was most associated with sulfa resistance of *P. jirovecii* in the current study?**

A. Need for mechanical ventilation at the time of PCP diagnosis

B. *P. jirovecii* type 10

C. *P. jirovecii* type 7

D. DHPS M2 mutation

**4. Which of the following variables was most associated with a higher risk of death due to PCP in the current study?**

A. DHPS M2 mutation

B. DHPS M3 mutation

C. *P. jirovecii* type 7

D. Older age

## Activity Evaluation

**1:** The activity supported the learning objectives.

Strongly Disagree				Strongly Agree
1	2	3	4	5
**2. The material was organized clearly for learning to occur.**				
Strongly Disagree				Strongly Agree
1	2	3	4	5
**3. The content learned from this activity will impact my practice.**				
Strongly Disagree				Strongly Agree
1	2	3	4	5
**4. The activity was presented objectively and free of commercial bias.**				
Strongly Disagree				Strongly Agree
1	2	3	4	5
